# Cytotoxicity Mechanism of Two Naphthoquinones (Menadione and Plumbagin) in *Saccharomyces cerevisiae*

**DOI:** 10.1371/journal.pone.0003999

**Published:** 2008-12-22

**Authors:** Frederico Augusto Vieira Castro, Diana Mariani, Anita Dolly Panek, Elis Cristina Araújo Eleutherio, Marcos Dias Pereira

**Affiliations:** Laboratório de Investigação de Fatores de Estresse (LIFE), Departamento de Bioquímica, Instituto de Química, Universidade Federal do Rio de Janeiro (UFRJ), Rio de Janeiro, Rio de Janeiro, Brasil; The Research Institute for Children at Children's Hospital New Orleans, United States of America

## Abstract

**Background:**

Quinones are compounds extensively used in studies of oxidative stress due to their role in plants as chemicals for defense. These compounds are of great interest for pharmacologists and scientists, in general, because several cancer chemotherapeutic agents contain the quinone nucleus. However, due to differences in structures and diverse pharmacological effects, the exact toxicity mechanisms exerted by quinones are far from elucidatation.

**Methodology/Principal Findings:**

Using *Saccharomyces cerevisiae*, we evaluated the main mechanisms of toxicity of two naphthoquinones, menadione and plumbagin, by determining tolerance and oxidative stress biomarkers such as GSH and GSSG, lipid peroxidation levels, as well as aconitase activity. The importance of glutathione transferases (GST) in quinone detoxification was also addressed. The GSSG/GSH ratio showed that menadione seemed to exert its toxicity mainly through the generation of ROS while plumbagin acted as an electrophile reacting with GSH. However, the results showed that, even by different pathways, both drugs were capable of generating oxidative stress through their toxic effects. Our results showed that the control strain, BY4741, and the glutathione transferase deficient strains (*gtt1Δ* and *gtt2Δ*) were sensitive to both compounds. With respect to the role of GST isoforms in cellular protection against quinone toxicity, we observed that the Gtt2 deficient strain was unable to overcome lipid peroxidation, even after a plumbagin pre-treatment, indicating that this treatment did not improve tolerance when compared with the wild type strain. Cross-tolerance experiments confirmed distinct cytotoxicity mechanisms for these naphthoquinones since only a pre-treatment with menadione was able to induce acquisition of tolerance against stress with plumbagin.

**Conclusions/Significance:**

These results suggest different responses to menadione and plumbagin which could be due to the fact that these compounds use different mechanisms to exert their toxicity. In addition, the Gtt2 isoform seemed to act as a general protective factor involved in quinone detoxification.

## Introduction

Quinones structurally encompass pigments, antibiotics, vitamin K and coenzymes. These compounds may represent a chemical defense for plants and have many known biological properties such as one-electron transfer agents in aerobic metabolism, therefore, they represent drugs extensively used in studies of oxidative stress [1]. They are of great toxicological and pharmacological interest, due to the fact that several cancer chemotherapeutic agents contain the quinone nucleus [1]–[3]. From a toxicological perspective, two major mechanisms have been proposed for the cytotoxic action of quinones, such as menadione (2-methyl-1,4-naphthoquinone: vitamin K3) and plumbagin (5-hydroxy-2-methyl-1,4-naphthoquinone) in a variety of biological systems. First, quinones undergo one electron reduction by enzymes such as microsomal NADPH-cytochrome P-450 reductase or mitochondrial NADH ubiquinone oxidoreductase, yielding the corresponding semiquinone radicals. Under aerobic conditions, the semiquinone radical participates in redox cycling to generate reactive oxygen species (ROS) like superoxide anion (O_2_^•^) and hydrogen peroxide (H_2_O_2_). Second, quinones are potent electrophiles, capable of reacting with the thiol groups of proteins and glutathione (GSH). In fact, generation of GS-conjugates catalyzed by glutathione tranferase isoforms (GST) with depletion of GSH has been associated with menadione-induced cytotoxicity and oxidative stress [2]–[5].

Oxidative stress is a process that can cause damage to membranes, proteins and also to DNA, which may induce apoptosis [6]. It has been reported that this process is related to the natural process of ageing and to diseases, such as atherosclerosis, cancer, Alzheimer and other neurodegenerative diseases [6]. This kind of stress can be generated endogenously by the aerobic way of life and/or exogenously by exposure to drugs [6]. Plumbagin is a naphthoquinone, a naturally occurring, yellow pigment produced by members of Plumbaginaceae, which accumulates mostly in roots. It has anti-cancer, leishmanicidal, anti-bacterial and anti-fungal activities, it can be applied topically for acne, inflammatory diseases, and ringworm, and is also effective against insects [1], [7]. On the other hand, menadione is a multivitamin component and a therapeutic agent for hypothrombinemia and cancer. Its cytotoxic mechanism has been associated with the excessive generation of ROS such as superoxide radicals, singlet oxygen, and hydrogen peroxide. Thus, it is a compound that has been widely used as a model for studies of oxidative damage [4], [8].

GSH is the most abundant non-protein thiol and a multifunctional intracellular antioxidant in cells. It consists of a tripeptide of glutamate, cysteine and glycine characterized by a reactive thiol group and γ-glutamyl bond. In addition, it is considered to be the major thiol-disulphide redox buffer of the cell, highly abundant in the cytosol, nuclei and mitochondria [9]. The main protective roles of GSH against oxidative stress are: its role as a cofactor of several detoxifying enzymes against oxidative stress, e.g. glutathione peroxidase (GPx), glutathione transferase (GST) and its role in regenerating the most important antioxidants, vitamins C and E back to their active forms [9], [10].

Glutathione transferases (GSTs) are enzymes which conjugate xenobiotics or their metabolites to glutathione (GSH), and they are also important in processes such as protection against oxidative stress, regulation of gene expression, or signal transduction [11], [12]. GSTs are divided into classes based on sequence, substrate specificity, or immunological properties and can be found in the cytosol as membrane-associated (microsomal) forms, as well as in organelles such as mitochondria or peroxisomes [11], [13], [14]. The yeast *Saccharomyces cerevisiae* harbors two proteins with GST activity, the products of the genes *GTT1* and *GTT2* [15], and three omega class GSTs (Gto1, Gto2, Gto3) with an enzyme activity similar to human omega GSTs hGTO1-1 and hGTO2-2 [13]. Among GST isoforms from *S. cerevisiae,* Gtt2 seems to be the most important in cadmium and menadione detoxification [12], [16].

The unicellular baker's yeast *Saccharomyces cerevisiae* is a well-documented eukaryote model for molecular and cellular biology studies such as detoxification pathways. Indeed, a large number of genes mediating the resistance to xenobiotics have been identified in yeast [17]. In this study we investigated the main cytotoxicity mechanism of two similar naphthoquinones, menadione and plumbagin, by studying GSH and GSSG levels as well as monitoring markers of oxidative stress. The involvement of isoform Gtt2 in quinone (plumbagin) detoxification was also a target of our study since our previously published work demonstrated that Gtt2 is responsible for generating the GS-menadione conjugate as a detoxification mechanism of resistance [12].

## Materials and Methods

### Yeast strains, media and growth conditions

*Saccharomyces cerevisiae* strains used in this work were the wild-type strain BY4741 (*MATa; his3Δ1; leu2Δ0; met15Δ0; ura3Δ0)* and two mutants *gtt1Δ* (Like BY4741 except *YIR038c::kanMX4*) and *gtt2Δ* (Like BY4741 except *YLL060c::kanMX4*) deficient in GST isoforms. Stocks of yeast strains were maintained on solid 2% YPD (1% yeast extract, 2% glucose, 2% peptone and 2% agar) in proper conditions to avoid the selection of petites or suppressors. In the case of mutant strains, the medium also contained 0.02% geneticine. For all experiments, cells were grown up to mid log phase (1.0 mg dry weight/mL) in liquid 2% YPD using an orbital shaker, at 28°C and 160 rpm, with the ratio of flask volume/medium of 5/1.

### Glutathione determination

Reduced glutathione (GSH) was determined spectrophotometrically, in neutralized trichloroacetic acid (10% TCA) extracts, before and after stress conditions, by following the glyoxylase catalyzed production of S-lactoyl-GSH at 240 nm. Glutathione disulfide (GSSG) was determined in the same cuvette by addition of NADPH and glutathione reductase, and then following the change in absorbance at 340 nm. The redox ratio was expressed as the ratio between GSSG and GSH contents [18], [19].

### Oxidative stress

Cells at the mid-log phase were directly exposed to severe oxidative stress conditions (7 μM plumbagin or 20 mM menadione) during 60 min at 28°C at 160 rpm or previously submitted to an adaptive treatment with 0.5 μM plumbagin or 0.5 mM menadione during 1 hour and then exposed to oxidant or submitted to a cross adaptive treatment with low concentrations of menadione (0.5 mM/1 h) or plumbagin (0.5 μM/1 h) and then exposed to 7 μM plumbagin and 20 mM menadione respectively during 1 hour.

### Tolerance and lipid peroxidation

Tolerance against stress conditions was analyzed by plating in triplicate on solidified YPD medium, after proper dilution. The plates were incubated at 28°C for 72 h and the colonies counted. Viability was determined before and after stress conditions, using cells adapted or not. Tolerance was expressed as percentage of survival.

Lipid peroxidation was measured by TBARS (thiobarbituric acid reactive species) method which detects malondialdehyde (MDA), a final product of lipid peroxidation [20].

### Aconitase activity

Cell extracts were obtained from 50 mg cells. Lysates were prepared with 1.0 g of glass beads by vortexing for 30 s followed by incubation on ice for 30 s, repeating the process eight times. Aconitase activity was determined spectrophotometrically by monitoring the formation of NADPH at 340 nm as described by Gardner et al. (1995) [21].

### Data analysis

The results represent the mean±standard deviation of at least three independent experiments. Statistical differences were tested using ANOVA followed by Tukey-Kramer multiple comparison test and for comparison between the means we used a t-student test. The latter denotes homogeneity between experimental groups at *P*<0.05. Different letters mean statistically different results.

## Results and Discussion

### Alteration on GSH contents after quinones stress conditions

GSH plays an important role in the defense against oxidative stress by protecting cells against effects of ROS mainly through detoxification mechanisms and the capacity to regenerate the most important cell antioxidants [9], [10]. This antioxidant function is directly associated with the redox state of the couple GSSG/GSH. Thus, accumulation of oxidized glutathione inside the cells is an excellent parameter to measure the levels of oxidative stress of an organism. It has been demonstrated that high GSSG concentrations may damage many enzymes oxidatively through the reaction of GSSG with protein sulphydryl groups. Also, the GSH level is an important factor in the protection against several stress conditions. Its protection originates from a multifactorial mechanism that involves detoxification and modulation of the cellular redox state and the subsequent redox-sensitive cell-signaling pathways which, in turn, interact with pro- and anti-apoptotic signals [22]. Due to these factors, low GSH levels can be associated with decrease in cell proliferation, protection against apoptosis and ROS elimination [9], [22].

To analyze whether menadione and plumbagin act as recycling agents or electrophiles, we determined the levels of GSH and GSSG as well as the GSSG/GSH ratio after exposure of cells to either 20 mM menadione or 7.0 μM plumbagin during 1 h. According to Table 1, while both drugs induced GSH depletion, menadione presented a higher capacity to mobilize GSH than plumbagin. The wild-type strain showed a greater increase in GSSG and in the GSSG/GSH ratio after a menadione stress. Interestingly, GSSG levels, after plumbagin stress, were similar to the non-stress condition. Under both stresses, the *gtt1Δ* strain showed a very similar level of GSH when compared to the wild-type. However, in this mutant, the increase in GSSG after menadione was lower, producing an enhancement of the GSSG/GSH ratio, 68% lower than that observed in the wild type (Table 1). Perhaps this result might be explained by the action of Gtt2 and/or Gto2, which are involved in quinone detoxification by conjugating with GSH. In fact, in cells growing exponentially on glucose, *GTT2* expression is higher in the *gtt1Δ* strain than in the wild type [12]. As can be seen in Table 1, in the *gtt2Δ* strain both quinones caused total depletion of GSH. On the other hand, the increase in the GSSG/GSH ratio after menadione stress, in the *gtt2Δ*, as well as in *gtt1Δ* mutant strains, was not comparable to the wild type. Again, our results point to another GST in *S. cerevisiae* that might be involved in cellular protection. In fact Gto2, recently characterized, was induced by oxidative stress [13]. Interestingly, the increase in GSSG observed after plumbagin stress could be related to the absence of the Gtt2 isoform that makes GSH available for the oxidation process. In the absence Gtt2, plumbagin generated the greatest increase in the GSSG/GSH ratio, which could be related to total GSH depletion not observed by the other strains, indicating that Gtt2 is involved in the detoxification process of plumbagin or oxidative stress products. Thus, these results suggest that although menadione and plumbagin are both naphthoquinones, they possess different mechanisms of exerting their toxicity in *S. cerevisiae* cells. While menadione seems to act mainly as a redox cycling agent generating ROS, the main mechanism of plumbagin seems to be involved in reacting with GSH as an electrophile with no generation of GSSG. However, the generation of a complex between plumbagin and GSH could also be associated with oxidative stress due to GSH mobilization. A number of different quinones with great structural differences have been shown to possess distinct mechanisms of action. While some induce the generation of ROS, others may act as electrophiles [5]. It is known that plumbagin treatment decreased viability of human prostate cancer cells due to apoptosis induction, which was accompanied by ROS generation and depletion of intracellular GSH levels [23]. However, the authors did not measure the GSSG profile. It has been reported by others that blocking GSH uptake increases GSSG and protein-bound SSG in mitochondria which is related to apoptosis activation [24]. In fact, controlled changes in GSSG/GSH redox potential are associated with functional state, varying with proliferation, differentiation and apoptosis. Our results demonstrate that a single addition of a hydroxyl group to the naphthoquinone nucleus is sufficient to modify the cytotoxicity mechanism due to increasing eletrophilic properties.

**Table 1:** Effect of menadione and plumbagin on glutathione.

Strains	Conditions	GSH	GSSG	GSSG/GSH Ratio
BY4741	Normal growth	30.0±2.3	16.9±1.9	0.6
	Menadione stressed	1.0±0.0*	80±0.3*	80.0
	Menadione treated and stressed	1.0±0.0*	25.4±2.9*	25.4
	Plumbagin stressed	9.6±0.9*	22.2±2.8*	2.3
	Plumbagin treated and stressed	1.0±0.0*	23.8±2.3*	23.8
*gtt1Δ*	Normal growth	29.9±9.0	21.6±2.8	0.7
	Menadione stressed	1.0±0.0*	25.5±0.2*	25.5
	Menadione treated and stressed	ND	ND	ND
	Plumbagin stressed	4.4±0.0*	19.4±3.1	4.4
	Plumbagin treated and stressed	ND	ND	ND
*gtt2Δ*	Normal growth	19.4±9.4	13.8±1.1	0.7
	Menadione stressed	1.0±0.0*	31.4±0.1*	31.4
	Menadione treated and stressed	ND	ND	ND
	Plumbagin stressed	1.0±0.0*	20.4±2.5*	20.4
	Plumbagin treated and stressed	ND	ND	ND

The GSH and GSSG levels were expressed in nmols/mg of cell. Normal Growth refers to a non-stress condition while menadione or plumbagin stressed refers to cells stressed with 20 mM menadione or 7.0 μM plumbagin for 1 h, respectively. Menadione and Plumbagin treated and stressed refers to cells pre-treated with 0.5 mM menadione or 0.5 μM plumbagin for 1 h before being stressed with the same drugs. The means obtained for drug treated cells were compared with the normal growth condition of each strain using a t-student test and ^*^ represents statistically different results at *P*<0.05.

ND–not determined.

It has been well documented that cells growing on glucose are hypersensitive to a sudden severe stress. However after mild stress conditions, cells can acquire tolerance against subsequent lethal stresses [12], [25]. We have previously demonstrated that a menadione pre-treatment leads to acquisition of tolerance against lethal menadione stress [12]. As shown in Table 1, determination of GSH and of the GSSG/GSH ratio after pre-treatments with menadione (0.5 mM/1 h) and plumbagin (0.5 μM/1 h), in the wild-type strain, indicate that only the menadione treatment was responsible for reducing the increase in the GSSG/GSH status, indicating that cells became more tolerant to oxidative stress, presumably due to increased levels of free GSH. These results suggest that treatment with menadione activates antioxidant defense mechanisms responsible for GSH production, or recycling, or by both processes, as previously published [25]. Interestingly, pre-treatment with plumbagin followed by lethal stress increases the redox ratio profile. This can be explained by the fact that the increase in the GSSG/GSH ratio was due to the reduced levels of GSH rather than to the increase of GSSG which, in fact, remained unchanged. In a previous work we observed that a *gsh1* mutant strain of *S. cerevisiae* did not acquire tolerance against menadione stress after treatment with a low menadione dosage [25]. In the same work we demonstrated that treatment with menadione was able to induce GSH synthesis with no modification in the antioxidant enzyme catalase activity [25]. In addition, it has been previously published that the Yap1 target-gene repertoire, that includes activities important for both maintenance of cellular redox homeostasis and ROS metabolism, and in cellular xenobiotic detoxification (ATP-binding cassettes transporters, GSH transferases, GSH biosynthetic pathway), is activated by xenobiotics such as menadione [26].

### Determination of oxidative stress levels and the involvement of Gtt2 isoform in cellular protection

**Figure 1:**
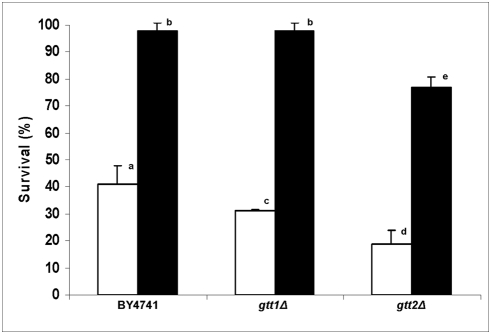
*S. cerevisiae* tolerance to plumbagin. Yeast cells harvested in mid log phase, were directly stressed with 7.0 μM plumbagin (white bars) or were previously treated with 0.5 μM plumbagin/1 h and then submitted to severe stress conditions (black bars). Tolerance was expressed as percentage of survival.

In our previous work we observed that menadione (20 mM/1 h) reduces the survival rates of BY4741 (50%) while for strains deficient in the Gtt isoforms, this stress was extremely lethal, showing the importance of these enzymes in the response to menadione stress [12]. In addition, we also observed that pre-treatment with menadione lead to acquisition of tolerance to a subsequent lethal stress in all strains studied, although the Gtt2 deficient strain continued to be the most affected [12]. Glutathione transferases are important components of the antioxidant defense system since they are involved in the formation of GS-conjugates with exogenous compounds such as quinones, or with products of lipid, protein and DNA oxidation, generated by oxidative stress, leading to less reactive products that are readily excreted [11]. Since plumbagin did not seem to be involved with direct generation of oxidative stress we expected that tolerance of yeast cells to this drug would exhibit a distinct profile. However, cells exposed to plumbagin (7.0 μM/1 h) presented a similar profile of tolerance as previously reported for menadione (Figure 1). Although both *gtt1Δ* and *gtt2Δ* strains showed an increased sensitivity to lethal stress, the deficiency in the Gtt2 isoform was more severe. This result also suggests the involvement of the Gtt2 isoform in the generation of GS-conjugates with plumbagin.

**Figure 2:**
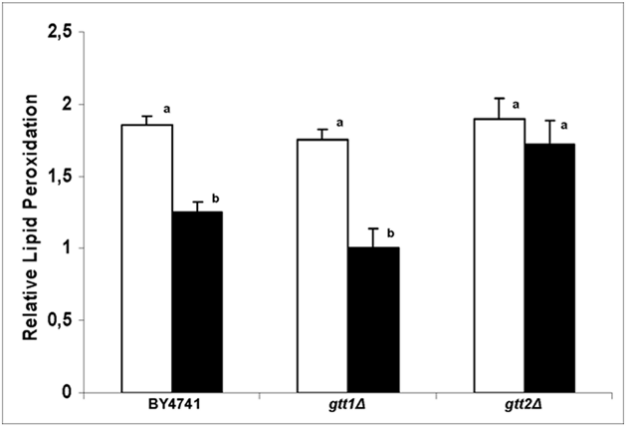
Enhancement of lipid peroxidation in wild-type and Gtt deficient cells caused by plumbagin. The increase in lipid peroxidation was expressed as a ratio between the levels of lipid peroxidation of plumbagin stressed and non-stressed cells. Yeast cells, harvested in mid log phase, were directly stressed with 7.0 μM plumbagin (white bars) or were previously treated with 0.5 μM plumbagin before been submitted to severe plumbagin stress (black bars).

In our previous study using menadione as an oxidant agent we observed that glutathione transferase 2 was involved with cellular protection due to detoxification and elimination of toxic products generated through oxidative stress [12]. Here, we also investigated the role of this GST isoform in protection and elimination of toxic products generated by plumbagin administration. Lipid peroxidation was increased after severe stress, in all strains used, (Figure 2). However, when a pre-treatment with plumbagin (0.5 μM) was applied, these levels decreased in the control strain as has been reported for a menadione stress [12]. On the other hand, lipid peroxidation in the adapted *gtt2* mutant remained high indicating that the Gtt2 isoform acts as a general protective protein against naphthoquinone toxicity (Figure 2). Interestingly, plumbagin was also able to promote oxidative stress through an indirect mechanism, by a nucleophilic attack on GSH, as seen by the increase in lipid peroxidation.

Due to the oxidative damage of lipids observed after the plumbagin stress, we decided to analyze the activity of aconitase, a four iron–four sulfur (4Fe-4S) cluster enzyme which is often used in studies of oxidative stress as a sensor of *in vivo* superoxide generation [27]. This enzyme is inactivated by superoxide through the removal of the labile iron atom. Aconitase is a good marker of oxidative stress caused by superoxide generation because it is not rate-limiting in this pathway and thus its activity can be substantially reduced without major effects on cellular metabolism [27]. Using exponential cells of the wild-type strain we found an aconitase activity of 52 mU/mg of protein under normal growth conditions with total inhibition of aconitase activity after administration of the drugs, even after previous menadione and plumbagin treatments, leading to reduction of lipid peroxidation and acquisition of tolerance. Thus, both quinones seem to generate an oxidative stress, albeit by different mechanisms.

### Plumbagin and menadione show distinct adaptive responses to stress

**Figure 3:**
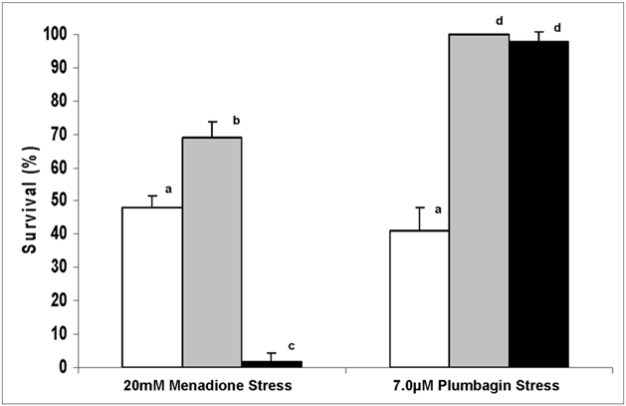
Determination of cellular response to naphthoquinones after cross-protection treatment. First exponential cells of the wild-type strain were submitted to a lethal stress either with 20 mM menadione or with 7.0 μM plumbagin (white bars), or were previously adapted with 0.5 mM menadione (gray bars) or 0.5 μM plumbagin (black bars).

Most studies concerning oxidative stress response have been focused on stress-inducible proteins and gene expression [28]. In fact, in *S. cerevisiae*, there is an overlap between stress responses induced by a variety of agents, such as superoxide anion and H_2_O_2_, and heat shock [25]. In this work, although both drugs showed the capacity to generate oxidative stress by increasing lipid peroxidation and abolishing aconitase activity, they showed different profiles in GSH mobilization. In order to investigate the mechanism of cellular response to the naphthoquinones, menadione and plumbagin, we carried out a cross-protection treatment to determine whether both drugs would induce the same defense systems which would confer tolerance to both menadione and plumbagin. Thus, *S. cerevisiae* cells were exposed either to 0.5 μM plumbagin/1 h and then exposed to 20 mM menadione/1 h or, to a pre-treatment with menadione (0.5 mM/1 h) before a lethal stress with plumbagin (7.0 μM/1 h). Unexpectedly, the plumbagin treatment did not induce acquisition of tolerance to a menadione stress in the wild-type strain, suggesting that signaling for cellular response or induction of protective genes to this naphthoquinone is different than to menadione. However, pre-treatment with menadione was sufficient to promote total tolerance to a plumbagin lethal stress, indicating that menadione confers plumbagin adaptation (Figure 3). Therefore, our results suggest that a menadione treatment would be responsible for activating gene expression contributing to an increase in tolerance to plumbagin. In addition, although plumbagin pre-treatment induced protection against a plumbagin lethal stress, this pre-treatment did not protect cells against the lethal effect of menadione. In fact, plumbagin treatment promoted an increase in sensibility to a menadione stress, presumably due to the previous GSH mobilization during pre-treatment, which then reduced the availability of GSH for protecting cells during a menadione stress.

Finally, these results suggest that cellular responses to these drugs are very different, strengthening the hypothesis that the toxicity mechanisms for both quinones are distinct. With respect to the role of Gtt2 in quinone toxicity, the results obtained support the importance of this isoform for detoxification of oxidative stress inducing drugs and by-products of oxidative damage.
